# A novel homozygous variant in PADI6 is associate with human cleavage-stage embryonic arrest

**DOI:** 10.3389/fgene.2023.1243230

**Published:** 2023-08-29

**Authors:** Guangyi Cao, Xiangyu Zhu, Yuling Lin, Junshun Fang, Xiaoyue Shen, Shanshan Wang, Na Kong

**Affiliations:** ^1^ Center for Reproductive Medicine and Obstetrics and Gynecology, Nanjing Drum Tower Hospital, Affiliated Hospital of Medical School, Nanjing University, Nanjing, China; ^2^ Center for Molecular Reproductive Medicine, Nanjing University, Nanjing, China; ^3^ State Key Laboratory of Reproductive Medicine and Offspring Health, Nanjing Medical University, Nanjing, China

**Keywords:** PADI6, mutation, cleavage arrest, embryos, reproduction

## Abstract

Repeated absence of useable embryos is a difficult problem for infertility patients. Among them, embryonic developmental arrest is more common, but the genetic cause is not known. The embryos of a patient who came to our hospital three times could not develop beyond the four-cell stage. In addition to recording the developmental details of the embryos by daily photo-taking, the *PADI6* ^R132C^ homozygous variants was further confirmed by whole-exome sequencing. Subsequently, *PADI6* ^R132C^ was analyzed by bioinformatics methods for conservativeness across species. In addition, the possible impact of the pathogenic mutation on the structure of the protein PADI6 were also assessed. Generally, we identified a homozygous variants [NM_207421.4, c.394C>T(p.R132C] in the middle protein-arginine deiminase domain in *PADI6* gene. The homozygous variant is highly conserved across species. Homozygous variant in *PADI6* ^R132C^ could cause a human cleavage-stage embryonic arrest in female patients. These findings provide further evidence for the important roles of the homozygous PADI6^R132C^ variant in embryonic development. Our findings contribute to a deeper understanding of the molecular genetic basis of female infertility.

## Introduction

Human reproduction begins with gametogenesis. High-quality sperm-oocyte union is followed by degradation of a large amount of maternal mRNA and protein in the oocyte. The embryonic genome is activated and the embryo begins to divide, passing through four-cell, eight-cell up to blastocyst. Failure of any step in this process results in infertility (Sang et al., 2021). The oocyte-to-embryo transition (OTE) process is initiated with the resumption of oocyte meiosis. This OTE process involves the initiation of degradation and translational activation of numerous unstable maternal mRNAs stored in the cytoplasm (Svoboda et al., 2015; Sha et al., 2019). Maternal factors have an important role in the OTE process. If OTE is faulty, even treatment with *in vitro* fertilization (IVF)/intracytoplasmic sperm injection (ICSI) cycles may make it difficult to obtain normally developed blastocysts.

The subcortical maternal complex (SCMC) is a key maternal factor that is distributed in the oocyte and embryonic cortex. The SCMC has been identified with well-defined components such as peptidylarginine deiminase, type VI (PADI6), maternal antigen that embryos require (MATER), factor located in oocytes permitting embryonic development (FLOPED), transducin-like enhancer of split 6 (TLE6), and KHDC3 (KH domain containing 3), among others(Bebbere et al., 2021). In clinical practice, early embryonic arrest is common and is one of the main reasons for failed IVF/ICSI attempts (Gardner and Lane, 1997). However, the exact cause of this genetic phenotype is not known. Novel mutations in *NLRP5*, *TLE6* and *KHDC3L* have been reported in the literature to cause preimplantation embryonic arrest in human (Alazami et al., 2015; Cao et al., 2018; Wang et al., 2018; Mu et al., 2019; Lin et al., 2020; Maddirevula et al., 2020; Xu et al., 2020; Sang et al., 2021; Zheng et al., 2021).

Female *Padi6* ^−/−^ mouse is infertile and developmental arrest occurs at the 2-cell stage. The mechanism of 2-cell arrest in *Padi6* ^−/−^ embryos may be due to failure to fully activate the embryonic genome. In *Padi6* ^−/−^ 2-cell embryos, the level of ribosomal components is reduced and *de novo* protein synthesis is dysregulated (Yurttas et al., 2008). Similarly, early embryonic developmental arrest was also found in patients with *PADI6* mutations (Xu et al., 2016). Phosphorylated RNA polymerase II and genes associated with embryonic genome activation were significantly reduced in embryos of patients with PADI6 mutations. This phenotype is consistent with the presence of impaired embryonic genome activation in *Padi6* ^−/−^ mice (Xu et al., 2016). In addition to the early embryonic arrest phenotype, PADI6 mutations in human also result in recurrent hydatidiform moles (Qian et al., 2018; Dong et al., 2022).

In this study, we identified by whole exome sequencing a homozygous variants [NM_207421.4, c.394C>T(p.R132C)] in the middle protein-arginine deiminase (PAD) domain in *PADI6* gene.PADI6 mutant embryos have impaired embryonic development and fail to develop beyond the 4-cell stage. The effect of this new mutation on the PADI6 protein was then further confirmed by bioinformatic analysis.

## Materials and methods

### Ethics approval

This study was approved by the Ethics Committee of Nanjing Drum Tower Hospital (2021-384-01). The embryos observed in this study were obtained from the Center for Reproductive Medicine, Drum Tower Hospital, School of Medicine, Nanjing University. Informed consent was obtained from the participants for the collection of clinical samples related to this experiment.

### Imaging

Bipronucleated (2PN) zygotes were selected separately and placed in separate culture drops using G1 culture medium (Vitrolife, Sweden). Each embryo was photographed using what DMi8 instrument (Leica, Germany).

### Whole-exome sequencing (WES) and variant analysis

Genomic DNA was extracted from the peripheral blood test samples provided by the subjects, and the DNA was firstly broken and libraries were prepared. The sequenced DNA sequences were compared with the human genome hg19 reference and the coverage and sequencing quality of the target regions were evaluated, and the variants were analyzed for bioinformatics and pathogenicity. The nomenclature of variants was referred to the nomenclature provided by the Human Genome Variation Society (HGVS) (http://varnomen.hgvs.org/). Criteria for grading pathogenic variants were developed according to the American College of Medical Genetics and Genomics standards and guidelines for the interpretation of variations (Richards et al., 2015; Kalia et al., 2017). Deletions and insertions (microvariations) within a range of 10 base pairs or less cannot detect potentially pathogenic variants in gene regulatory regions and deep intronic regions. Our method is not suitable for detecting special types of variations, such as dynamic mutations, large segment deletions or duplications, complex recombinations, and genomic structural variations (e.g., inversions, translocations, rearrangements).

### Model drawing and conservativeness analysis

Schematic structure of the mutated PADI6 protein sites was drawn using IBS 2.0 (Xie et al., 2022). Conserved PADI6 amino acids in different species such as human, mouse, monkey, pig, sheep and rat were analyzed using the Align function of UniProt website (https://www.uniprot.org/). WT and mutated PADI6 [NM_207421.4, c.394C>T(p.R132C)] were schematically plotted using SWISS-MODEL software (https://swissmodel.expasy.org) and the control template chosen was 4dkt.1.B.pdb.

### Dynamic expression of PADI6

The dynamics of PADI6 mRNA in different species at embryonic periods (2PN, 2-cell, 4-cell, 8-cell, morula, early-ICM, late-ICM) was reanalyzed according to the single-cell transcriptome database (Boroviak et al., 2018). Dynamic expression of ribosome-bound *Padi6* mRNA in mouse oocytes and embryos was reanalyzed according to the mRNA translatomics database (Xiong et al., 2022).

## Results

### Phenotype of patients with the PADI6^R132C^ mutations

**FIGURE 1:**
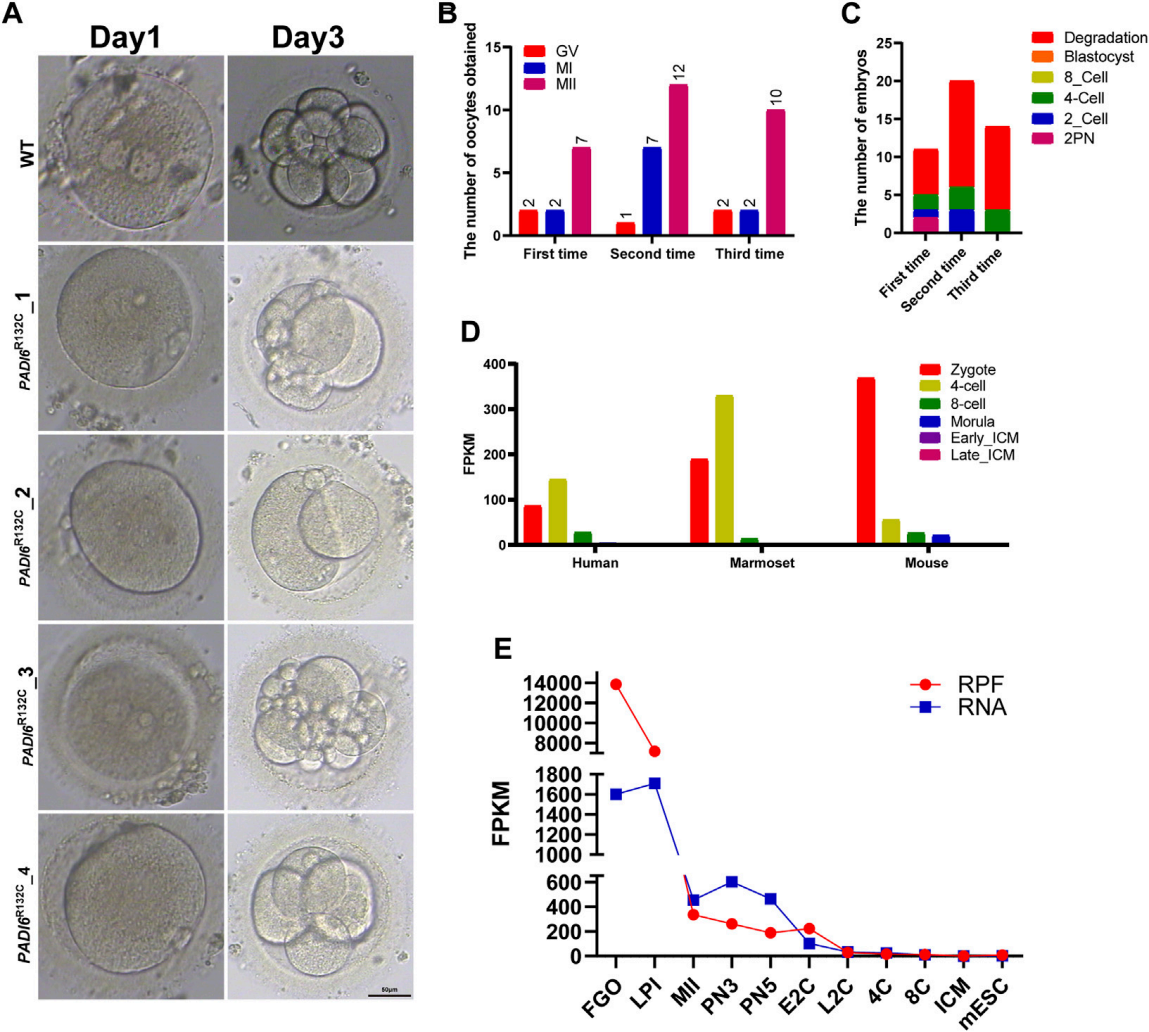
Morphological characteristics of mutant PADI6 embryos and dynamic expression of PADI6 in different species. **(A)** Representative images of wild-type and mutant PADI6 embryos were simulated. As shown in the figure, at day3 PADI6 wild-type embryos can develop up to 8-cells, but PADI6 mutant embryos cannot continue to develop. The scale bar is 50μm. **(B)** The number and period of oocytes obtained from three oocyte retrievals in this patient. **(C)** The final outcome statistics of the embryos obtained from three IVF or ICSI procedures in this patient. As shown in the figure, this patient’s embryos developed up to the 4-cell stage and could not continue their development. **(D)** Single-cell RNA-sequencing (scRNA-seq) transcriptome showed *PADI6* mRNA dynamics from zygote to ICM in mouse, marmoset and human. **(E)** Dynamic ribosome-bound RNA expression (RPF) changes of *Padi6* mRNA from the oocyte to the ICM in mouse. RPF folding line chart represents RNA changes bound to ribosomes using low-input Ribo-seq (Ribo-lite). RNA folding line chart represents regular mRNA sequencing (mRNA-seq). RPF, ribosome protected fragment; Fully grown oocytes, FGOs; LPI, late prometaphase I; MII, metaphase II; PN3, early one-cell stage; PN5, late one-cell stage; E2C, early two-cell stage; L2C, late two-cell stage, 4C, four-cell stage; 8C, eight-cell stage, ICM, inner cell mass of blastocyst; mESC, mouse embryonic stem (mES) cells.

A 28-year-old patient presented at our center with primary infertility. After 6 years of marriage, she was diagnosed with primary infertility. Ultrasonography revealed normal uterine and ovarian morphology, with observable mature follicles. Her hormonal profile demonstrated normal levels of follicle-stimulating hormone (FSH) and anti-Müllerian hormone (AMH). Interestingly, in this patient, oocytes were retrieved on three separate occasions, and the metaphase II (MII) stage oocytes obtained numbered 7, 12, and 10, respectively. It is noteworthy that almost 40% of the oocytes did not develop into the MII stage during these three retrieval cycles (results: 4 out of 11 in the first cycle; 8 out of 20 in the second cycle; 4 out of 14 in the third cycle) (Figure 1B). Subsequently, these oocytes degenerated substantially, suggesting a critical role for PADI6 in oocyte maturation.

Although a reasonable number of MII oocytes were obtained, less than 20% of the PADI6^R132C^ mutants were able to develop to 4-cells (Figure 1C). After each fertilization of these oocytes (IVF or ICSI), the fertilized embryos could not develop beyond the third day of four-cell stage (Figures 1A, C). Routine semen analysis of their husbands showed normal fertility.

### Expression of human *PADI6*

Single-cell transcriptome data showed that *PADI6* gene was highly expressed in human 2PN embryos and 4-cell stages, but the expression value decreased rapidly in the 8-cell period. Interestingly, *PADI6* had a consistent expression pattern with human in Marmoset. Similarly, *Padi6* expression levels were highest in mouse 2PN embryos, but began to decrease rapidly in the subsequent 4-cells (Figure 1D) (Boroviak et al., 2018). To further characterize the dynamics of *Padi6* during oocyte meiotic maturation and preimplantation embryo development, using ribosome profiling (low-input Ribo-seq) revealed a significantly top level in translation efficiency of *Padi6* during the fully grown oocyte (FGO) in mouse (Figure 1E) (Xiong et al., 2022). These results indicate that *PADI6* expression is conserved across species and is abundant in 2PN embryo, suggesting an important role for PADI6 in zygote.

### Impact of PADI6^R132C^ mutations

**FIGURE 2:**
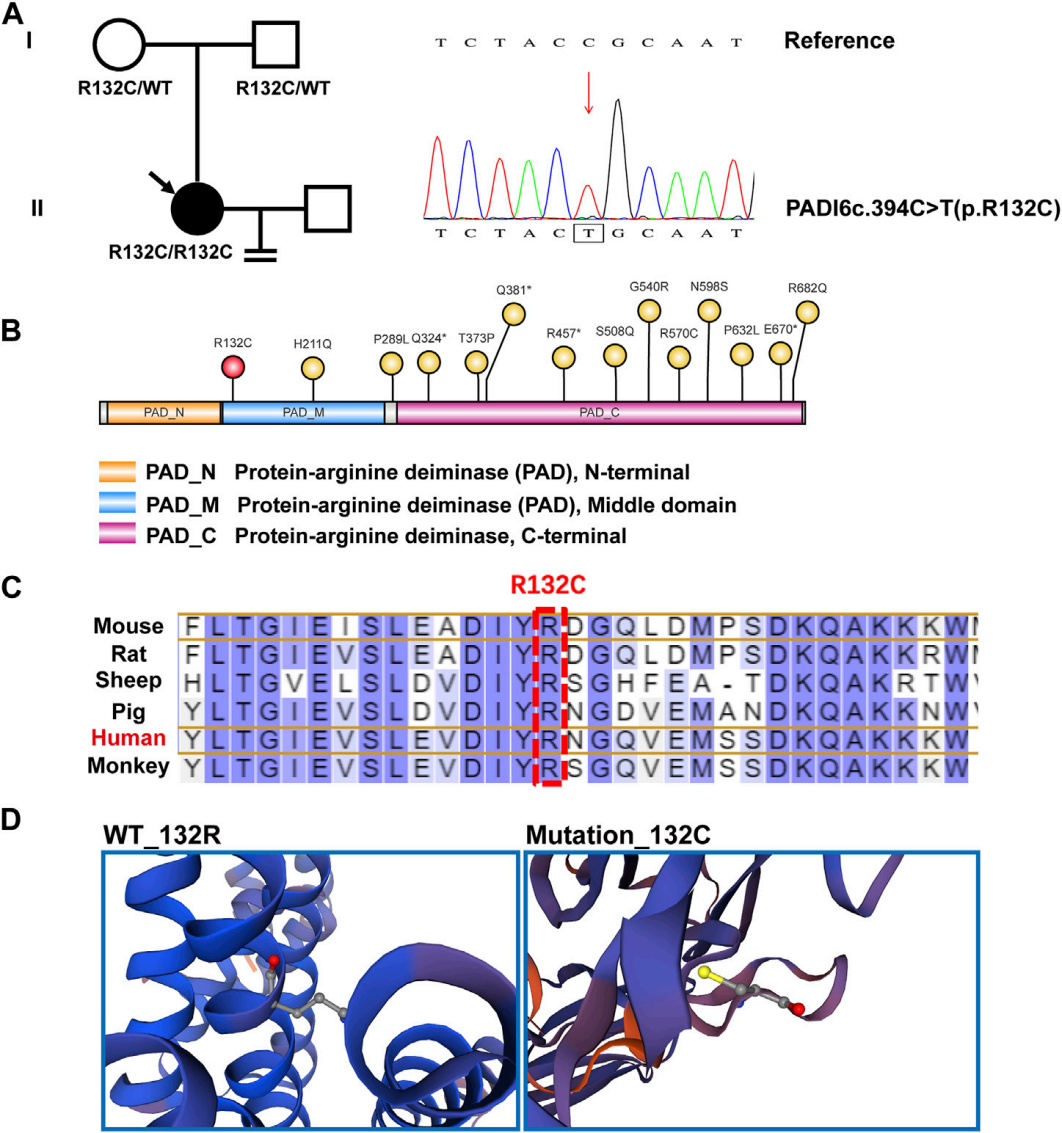
Genealogy and bioinformatics analysis of the proband. **(A)** The patient’s family is shown on the left, with arrows indicating the proband. The mutant PADI6 loci by whole-exome sequencing and hg19 reference genome comparison map, with a homozygous PADI6 variant (NM_207421.4, c.394C>T(p.R132C)), is shown in the right figure. **(B)** Indicative map of mutant sites in the structural domain of the protein PADI6. Yellow is the already reported sites and red is the new mutant sites R132C identified in this study. PAD_N, Protein-arginine deiminase N-terminal. PAD_M, Protein-arginine deiminase Middle domain. PAD_C, Protein-arginine deiminase C-terminal domain. **(C)** The residues R132 of PADI6 are highly conserved across six species. Human sites are marked in red, and R132 sites are marked with boxes. **(D)** Spatial structure pattern maps of wild-type and mutant PADI6 proteins were simulated using SWISS-MODEL software, revealing that the R132C variant alters the shape of the protein.

The PADI6^R132C^ mutations are localized in the protein-arginine deiminase (PAD) middle domain, which is a relatively rare site. Most of the reported mutated sites (12/14) are in the PAD_ C-terminal domain (Figure 2A, 2B) (Zheng et al., 2020). The amino acids of the PADI6^R132C^ mutations were found to be highly conserved by protein comparative analysis of six species from mouse, rat, sheep, pig, monkey to human (Figure 2C). Based on the predicted 3D spatial structure of the protein, PADI6^WT^ and PADI6^R132C^ mutations differed significantly in amino acid residues at position 132 (Figure 2D).

## Discussion

*PADI6* (peptidylarginine deiminase, type VI), an important maternal gene, is exceptionally abundantly expressed in meiotic maturation of oocytes (Figures 1D, E) (Xiong et al., 2022). By re-analyzing single-cell transcriptome sequencing data during human, marmoset and mouse pre-implantation embryonic development, we found that *PADI6* expression pattern was conserved across species (Figure 1C) (Boroviak et al., 2018). PADI6 is predominantly distributed in the subcortical region in oocyte and zygote, and is a major member of the subcortical maternal complex (SCMC) (Bebbere et al., 2021). The formation of specific fibrous structures, cytoplasmic lattices (CPLs), in the oocyte is inseparable from PADI6 (Esposito et al., 2007). Oocyte cytoplasmic lattices (CPLs) presumably provide ribosomal reservoirs for early embryos. In *Padi6*-KO oocytes, the ribosomes rRNAs associated with CPLs are significantly reduced. PADI6 can interact with MSY2 to preserve ribosomes and mRNAs in an inactive form. A simple but powerful mechanism is provided to ensure the successful activation of the embryonic genome by simultaneous release of mRNA and ribosomes (Yu et al., 2001; Yu et al., 2002; Liu et al., 2017).

In our findings, it was observed that approximately 40% of the immature oocytes obtained from the patient during the three retrieval cycles failed to reach maturity and subsequently degenerated. This observation is consistent with the role of PADI6 as a major component of the subcortical maternal complex (SCMC) in regulating oocyte meiosis. While there have been studies on the impact of PADI6 on oocyte meiosis in mice, further exploration is required to understand its role in human oocytes, given the limited availability of human samples for research purposes.

To date, a total of 14 mutant loci have been reported for the human PADI6 gene. Interestingly, twelve of these loci are found within the PAD_ C-terminal domain (12/14), while the novel loci we have newly identified are distinct, relatively rare, and primarily situated in the PAD_ Middle domain. These newly identified loci, being in close proximity to the N-terminal region of the PADI6 protein, have the potential to exert earlier effects on the protein’s functionality. Specifically, the mutation at the identified locus leads to a substitution of arginine with cysteine at position 132 in the PADI6 protein. The fundamental difference between cysteine and arginine lies in their respective R-groups. Arginine possesses a basic R-group, while cysteine exhibits a polar and neutral R-group. Cysteine is nearly insoluble in water, whereas arginine is readily soluble. Furthermore, our structural prediction analysis indicates that this amino acid mutation further impacts the spatial conformation of the protein.

Patients with *PADI6* mutation showed early embryonic arrest, zygotic cleavage failure, recurrent hydatidiform moles and other different phenotypes (Alazami et al., 2015; Wang et al., 2018; Mu et al., 2019; Lin et al., 2020; Maddirevula et al., 2020; Xu et al., 2020; Sang et al., 2021; Zheng et al., 2021; Dong et al., 2022). The different phenotypes of patients with PADI6 mutation are presumed to be related to the location of the PADI6 mutation (Qian et al., 2018). If one aims to rescue this phenotype, it could be attempted to repair the mutation using base editing gene-editing technology (McAuley et al., 2023). Additionally, supplementation of *PADI6*-mRNA could be explored as a potential rescue strategy. However, it is important to consider ethical considerations and engage in further discussions regarding the feasibility and implications of these ideas.

Mutations in two other members of the SCMC complex, NLRP7 and KHDC3L, contribute to 60% of the clinically recurrent hydatidiform moles (Murdoch et al., 2006; Parry et al., 2011). Immunofluorescence showed co-localization of PADI6 and NLRP7 (Qian et al., 2018). Although most mutated PADI6 proteins cause early embryonic developmental disorders, two variants (c.1793A>G, p.(Asn598Ser) and c.2045G > A, p.(Arg682Gln) caused recurrent hydatidiform moles. This result suggests that part of the function of PADI6 is still in play and that there may be some interaction between PADI6 and NLRP7 (Qian et al., 2018). In addition, in hydatidiform moles, PADI6 can regulate trophoblast cell migration-invasion through Hippo/YAP1 Pathway(Huang et al., 2021).

During Oocyte-to-Embryo transition (OTE) process, mouse embryonic genome activation starts from 2-cell stage and in humans from 4-cell stage (Eckersley-Maslin et al., 2018). In *Padi6*_KO mouse, two-cell embryos present impaired *de novo* protein synthesis and impaired embryonic genomic activation (Yurttas et al., 2008). Similarly, the majority of human embryos with *PADI6* mutations are arrest in four-cells, also suggesting abnormalities in human embryonic genome activation. It is interesting to follow whether new methods can be used to rescue the process of embryonic genome activation malfunction.

Although we observed the significant role of PADI6^R132C^ mutations in early embryonic development, this study does have some limitations. Firstly, the sample size is relatively small, and it would be necessary to identify the same mutation site with similar phenotypes in multiple reproductive centers. Secondly, due to the inherent limitations of the exome sequencing analysis process and the continuous refinement of the pathogenic variants database, our study may have missed the discovery of some other pathogenic variants in different genes (Richards et al., 2015; Kalia et al., 2017).

To conclude, our study identified a new mutated site of PADI6 especially at the PAD_middle domain, which enriches the understanding of PADI6 mutations causing early embryonic division arrest. Our findings improve the understanding of the genetic basis of female infertility.

## Data Availability

The original data presented in the study may be found in the article/Supplementary Material. Further inquiries can be directed to the corresponding authors.

## References

[B1] Alazami A. M. Awad S. M. Coskun S. Al-Hassan S. Hijazi H. Abdulwahab F. M. (2015). TLE6 mutation causes the earliest known human embryonic lethality. Genome Biol. 16, 240. 10.1186/s13059-015-0792-0 26537248 PMC4634911

[B2] Bebbere D. Albertini D. F. Coticchio G. Borini A. Ledda S. (2021). The subcortical maternal complex: emerging roles and novel perspectives. Mol. Hum. Reprod. 27, gaab043. 10.1093/molehr/gaab043 34191027

[B3] Boroviak T. Stirparo G. G. Dietmann S. Hernando-Herraez I. Mohammed H. Reik W. (2018). Single cell transcriptome analysis of human, marmoset and mouse embryos reveals common and divergent features of preimplantation development. Development 145, dev167833. 10.1242/dev.167833 30413530 PMC6240320

[B4] Cao G. Y. Li M. Z. Wang H. Shi L. Y. Su Y. Q. (2018). Interference with the C-terminal structure of MARF1 causes defective oocyte meiotic division and female infertility in mice. J. Biomed. Res. 32, 58–67. 10.7555/JBR.32.20170108 29353819 PMC5956259

[B5] Dong J. Fu J. Yan Z. Li L. Qiu Y. Zeng Y. (2022). Novel biallelic mutations in PADI6 in patients with early embryonic arrest. J. Hum. Genet. 67, 285–293. 10.1038/s10038-021-00998-8 34987164

[B6] Eckersley-Maslin M. A. Alda-Catalinas C. Reik W. (2018). Dynamics of the epigenetic landscape during the maternal-to-zygotic transition. Nat. Rev. Mol. Cell Biol. 19, 436–450. 10.1038/s41580-018-0008-z 29686419

[B7] Esposito G. Vitale A. M. Leijten F. P. Strik A. M. Koonen-Reemst A. M. Yurttas P. (2007). Peptidylarginine deiminase (PAD) 6 is essential for oocyte cytoskeletal sheet formation and female fertility. Mol. Cell Endocrinol. 273, 25–31. 10.1016/j.mce.2007.05.005 17587491

[B8] Gardner D. K. Lane M. (1997). Culture and selection of viable blastocysts: A feasible proposition for human IVF? Hum. Reprod. Update 3, 367–382. 10.1093/humupd/3.4.367 9459282

[B9] Huang B. Zhao Y. Zhou L. Gong T. Feng J. Han P. (2021). PADI6 regulates trophoblast cell migration-invasion through the hippo/YAP1 Pathway in hydatidiform moles. J. Inflamm. Res. 14, 3489–3500. 10.2147/JIR.S313422 34326657 PMC8314932

[B10] Kalia S. S. Adelman K. Bale S. J. Chung W. K. Eng C. Evans J. P. (2017). Recommendations for reporting of secondary findings in clinical exome and genome sequencing, 2016 update (ACMG SF v2.0): A policy statement of the American College of medical genetics and genomics. Genet. Med. 19, 249–255. 10.1038/gim.2016.190 27854360

[B11] Lin J. Xu H. Chen B. Wang W. Wang L. Sun X. (2020). Expanding the genetic and phenotypic spectrum of female infertility caused by TLE6 mutations. J. Assist. Reprod. Genet. 37, 437–442. 10.1007/s10815-019-01653-0 31897846 PMC7056779

[B12] Liu X. Morency E. Li T. Qin H. Zhang X. Zhang X. (2017). Role for PADI6 in securing the mRNA-MSY2 complex to the oocyte cytoplasmic lattices. Cell Cycle 16, 360–366. 10.1080/15384101.2016.1261225 27929740 PMC5324759

[B13] Maddirevula S. Awartani K. Coskun S. AlNaim L. F. Ibrahim N. Abdulwahab F. (2020). A genomics approach to females with infertility and recurrent pregnancy loss. Hum. Genet. 139, 605–613. 10.1007/s00439-020-02143-5 32172300

[B14] McAuley G. E. Yiu G. Chang P. C. Newby G. A. Campo-Fernandez B. Fitz-Gibbon S. T. (2023). Human T cell generation is restored in CD3δ severe combined immunodeficiency through adenine base editing. Cell 186, 1398–1416.e23. 10.1016/j.cell.2023.02.027 36944331 PMC10876291

[B15] Mu J. Wang W. Chen B. Wu L. Li B. Mao X. (2019). Mutations in NLRP2 and NLRP5 cause female infertility characterised by early embryonic arrest. J. Med. Genet. 56, 471–480. 10.1136/jmedgenet-2018-105936 30877238

[B16] Murdoch S. Djuric U. Mazhar B. Seoud M. Khan R. Kuick R. (2006). Mutations in NALP7 cause recurrent hydatidiform moles and reproductive wastage in humans. Nat. Genet. 38, 300–302. 10.1038/ng1740 16462743

[B17] Parry D. A. Logan C. V. Hayward B. E. Shires M. Landolsi H. Diggle C. (2011). Mutations causing familial biparental hydatidiform mole implicate c6orf221 as a possible regulator of genomic imprinting in the human oocyte. Am. J. Hum. Genet. 89, 451–458. 10.1016/j.ajhg.2011.08.002 21885028 PMC3169823

[B18] Qian J. Nguyen N. M. P. Rezaei M. Huang B. Tao Y. Zhang X. (2018). Biallelic PADI6 variants linking infertility, miscarriages, and hydatidiform moles. Eur. J. Hum. Genet. 26, 1007–1013. 10.1038/s41431-018-0141-3 29693651 PMC6018785

[B19] Richards S. Aziz N. Bale S. Bick D. Das S. Gastier-Foster J. (2015). Standards and guidelines for the interpretation of sequence variants: A joint consensus recommendation of the American College of medical genetics and genomics and the association for molecular pathology. Genet. Med. 17, 405–424. 10.1038/gim.2015.30 25741868 PMC4544753

[B20] Sang Q. Zhou Z. Mu J. Wang L. (2021). Genetic factors as potential molecular markers of human oocyte and embryo quality. J. Assist. Reprod. Genet. 38, 993–1002. 10.1007/s10815-021-02196-z 33895934 PMC8190202

[B21] Sha Q. Q. Zhang J. Fan H. Y. (2019). A story of birth and death: mRNA translation and clearance at the onset of maternal-to-zygotic transition in mammals†. Biol. Reprod. 101, 579–590. 10.1093/biolre/ioz012 30715134

[B22] Svoboda P. Franke V. Schultz R. M. (2015). Sculpting the transcriptome during the oocyte-to-embryo transition in mouse. Curr. Top. Dev. Biol. 113, 305–349. 10.1016/bs.ctdb.2015.06.004 26358877

[B23] Wang X. Song D. Mykytenko D. Kuang Y. Lv Q. Li B. (2018). Novel mutations in genes encoding subcortical maternal complex proteins may cause human embryonic developmental arrest. Reprod. Biomed. Online 36, 698–704. 10.1016/j.rbmo.2018.03.009 29606347

[B24] Xie Y. Li H. Luo X. Li H. Gao Q. Zhang L. (2022). IBS 2.0: an upgraded illustrator for the visualization of biological sequences. Nucleic Acids Res. 50, W420–W426. 10.1093/nar/gkac373 35580044 PMC9252815

[B25] Xiong Z. Xu K. Lin Z. Kong F. Wang Q. Quan Y. (2022). Ultrasensitive Ribo-seq reveals translational landscapes during mammalian oocyte-to-embryo transition and pre-implantation development. Nat. Cell Biol. 24, 968–980. 10.1038/s41556-022-00928-6 35697785

[B26] Xu Y. Qian Y. Liu Y. Wang Q. Wang R. Zhou Y. (2020). A novel homozygous variant in NLRP5 is associate with human early embryonic arrest in a consanguineous Chinese family. Clin. Genet. 98, 69–73. 10.1111/cge.13744 32222962

[B27] Xu Y. Shi Y. Fu J. Yu M. Feng R. Sang Q. (2016). Mutations in PADI6 cause female infertility characterized by early embryonic arrest. Am. J. Hum. Genet. 99, 744–752. 10.1016/j.ajhg.2016.06.024 27545678 PMC5010645

[B28] Yu J. Hecht N. B. Schultz R. M. (2001). Expression of MSY2 in mouse oocytes and preimplantation embryos. Biol. Reprod. 65, 1260–1270. 10.1095/biolreprod65.4.1260 11566752

[B29] Yu J. Hecht N. B. Schultz R. M. (2002). RNA-binding properties and translation repression *in vitro* by germ cell-specific MSY2 protein. Biol. Reprod. 67, 1093–1098. 10.1095/biolreprod67.4.1093 12297523

[B30] Yurttas P. Vitale A. M. Fitzhenry R. J. Cohen-Gould L. Wu W. Gossen J. A. (2008). Role for PADI6 and the cytoplasmic lattices in ribosomal storage in oocytes and translational control in the early mouse embryo. Development 135, 2627–2636. 10.1242/dev.016329 18599511 PMC2708103

[B31] Zheng W. Chen L. Dai J. Dai C. Guo J. Lu C. (2020). New biallelic mutations in PADI6 cause recurrent preimplantation embryonic arrest characterized by direct cleavage. J. Assist. Reprod. Genet. 37, 205–212. 10.1007/s10815-019-01606-7 31664658 PMC7000584

[B32] Zheng W. Hu H. Dai J. Zhang S. Gu Y. Dai C. (2021). Expanding the genetic and phenotypic spectrum of the subcortical maternal complex genes in recurrent preimplantation embryonic arrest. Clin. Genet. 99, 286–291. 10.1111/cge.13858 33020905

